# Association of Physical Performance and Pain With Fear of Falling Among Community—Dwelling Japanese Women Aged 65 Years and Older

**DOI:** 10.1097/MD.0000000000001449

**Published:** 2015-09-04

**Authors:** Yoshihito Tomita, Kazuhiko Arima, Mitsuo Kanagae, Takuhiro Okabe, Satoshi Mizukami, Takayuki Nishimura, Yasuyo Abe, Hisashi Goto, Itsuko Horiguchi, Kiyoshi Aoyagi

**Affiliations:** From the Department of Public Health, Nagasaki University Graduate School of Biomedical Sciences, Nagasaki, Japan (YT, KA, MK, TO, TN, YA, KA); Department of Rehabilitation, Nishi-Isahaya Hospital, Isahaya, Japan (YT, MK, TO, SM); Goto Health Care Office, Nagasaki, Japan (HG); and Center for Public Relations Strategy, Nagasaki University, Nagasaki, Japan (IH).

## Abstract

Our aim was to explore the association of physical performance and pain with fear of falling among community-dwelling Japanese women.

The subjects were 278 women aged 65 years and over. We collected information on fear of falling, painful joints, comorbidities, falls in the previous year, and cataracts. Walking time (distance of 6 m), chair stand time (5 times), grip strength, the timed up and go test (TUG), and functional reach were measured.

The prevalence of fear of falling was 36.3%, and it increased with age, but it was not significant (*P* = 0.081). Multivariate logistic regression analysis showed that poor physical performance (longer walking time, longer chair stand time, weaker grip strength, and longer TUG) and pain (low back, and upper and lower extremity pain) were significantly associated with fear of falling after adjusting for age, body mass index, comorbidities, falls in the previous year, and cataracts.

Maintaining physical functioning and managing pain may be important for elderly women with fear of falling.

## INTRODUCTION

Fear of falling is a major health problem among elderly people living in communities.^1^ Fear of falling is associated with reduced levels of physical activity,^2^ reduced ability to perform activities of daily living,^3,4^ increased risk of admission to an aged care institution,^4^ and decreased quality of life.^5,6^ It is common for fear of falling to occur after falls,^7^ but it can also occur without a history of falls.^8^ Previous studies have shown that fear of falling is affected by several factors such as gender, age, comorbidity, body pain, history of falls, visual impairment, depression, and cognitive function.^9–11^

Although the relationship between fear of falling and physical performance has been established,^9,12–16^ few studies have focused on elderly residents in Japan.^14^ Furthermore, although pain is reported to be associated with fear of falling,^14,17,18^ most studies have investigated general body pain, whereas few have focused on pain in specific areas such as the low back.^17,19^ The objective of the present study was to explore the association of physical performance and pain with fear of falling among community-dwelling Japanese women.

## SUBJECTS AND METHODS

This study was performed as a part of the Oshima Health Study, which was an investigation of the health status in community-dwelling residents. Details are described elsewhere.^20^ The participants of this study were 313 community-dwelling women aged ≥65 years, who were non-institutionalized and lived independently in Oshima Town, Nagasaki Prefecture, Japan. The health status investigation was performed in the period from 2001 to 2003 at the Oshima Health Center. All participants had sufficient cognitive functioning to answer the questionnaire.

In order to assess the prevalence of fear of falling, participants were asked whether they feel fear of falling using a question, “Are you afraid of falling?” Self-administered questionnaires were handed, which include the information on painful joints, comorbidities, falls in the previous year, and cataracts. The information on painful joints was collected with a questionnaire: “Which joints are currently painful or have been painful most days for at least the past month?” Participants were answered using a sketch of the skeleton. The answers of painful joints were categorized. For instance, the shoulders, elbows, wrists, and hand/finger joints were categorized into upper extremities; the hips, knees, ankles, and foot joint were categorized into lower extremities. The comorbidity data including heart disease, lung disease, stroke, or diabetes mellitus were collected.

Height (m) and weight (kg) were measured with the subject in light clothing and without shoes, and body mass index (BMI) was calculated as weight/height squared (kg/m^2^).

Walking time was quantified as the time of walking a 6-m distance with the subject's usual walking speed. Chair stand time was quantified as the time of standing up from a standard chair and sitting down in 5 times, without the assistance of their arms. These measured times were calculated into the average of two results. Grip strength was measured using a hydraulic hand dynamometer (Jamar hydraulic hand dynamometer; Jafayette Instrument Company, Inc., Jafayette, IN). The better performance from two trials used their dominant hand was deemed as the result. Timed up and go test (TUG) involves standing from a chair, walking 3 m, turning around, walking back to the chair, and sitting down. Subjects were instructed to complete the task at their usual walking speed. The TUG was quantified as the time of the full task, calculated as the average of 2 results. The functional reach test was quantified as the difference between the initial point (standing comfortably upright, facing forward, hand in a first, with the arm extended) and the reaching point (reaching forward as far as possible) without stepping or losing balance. These measured distances were calculated into the average of 3 results.

All subjects provided written informed consent to participate in the study before the health status examination. This study was approved by the Oshima Local Ethics Committee.

### Statistical Analysis

Women with missing values for any variables were excluded from analysis (n = 35), leaving 278 women for the final data analysis. Comparisons of prevalence of fear of falling by age group, and physical performance or pain between subjects with and without fear of falling were performed using the Student's *t*-test for continuous variables or the chi-square test for nominal variables. The association between fear of falling and physical performance measures or pain was assessed using logistic regression analysis, adjusting for age (model 1). As age, BMI, comorbidities, falls in the previous year, and cataracts may be associated with fear of falling, we included all the variables in another model (model 2). The Hosmer–Lemeshow test was used to evaluate the difference between observed and predicted prevalence in our multivariate logistic regression analysis. Odds ratios (OR) and 95% confidence intervals (CI) were calculated. A probability value of *P* *<* 0.05 was considered to indicate significance. All statistical analyses were performed using SPSS software version 21 (SPSS Inc., Chicago, IL).

## RESULTS

The characteristics of the subjects are shown in Table 1. Mean age and BMI were 72.6 years old and 23.5 kg/m^2^, respectively. Of the women, 22% had low back pain, 21% had upper extremity pain, and 37% had lower extremity pain. Twenty-eight percent of the women had at least 1 comorbidity, and 23% reported a history of falls in the previous year.

**TABLE 1:**
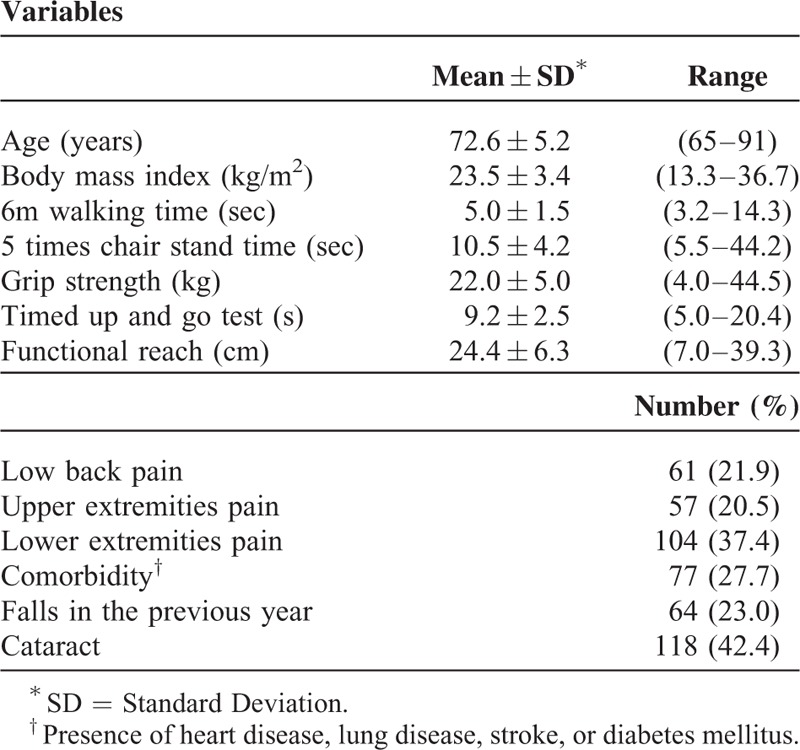
Subject Characteristics (n = 278)

The prevalence of fear of falling in all subjects was 36.3% (101/278). Table 2 shows the prevalence of fear of falling according to the age group. The prevalence of fear of falling in the > 75 years age group was higher than that of the 65 to 74 years age group, but it was not significant (*P* = 0.081).

**TABLE 2:**
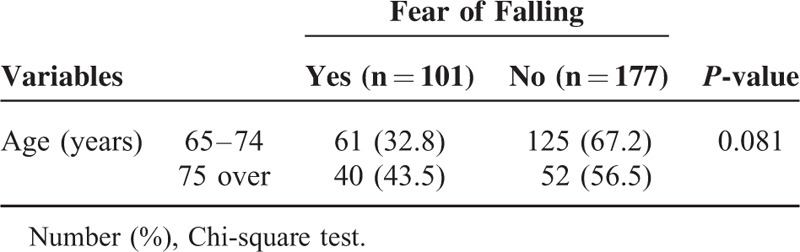
Prevalence of Fear of Falling by Age Group

Table 3 shows a comparison of physical performance or pain between women with fear of falling and those without. Women with fear of falling had poor physical functioning (longer 6-m walking time, longer five-time chair stand time, weaker grip strength and longer TUG) and higher prevalence of pain (low back pain, upper extremity pain and lower extremity pain), compared with women without fear of falling (*P* < 0.05). There was not a significant difference in functional reach among them.

**TABLE 3:**
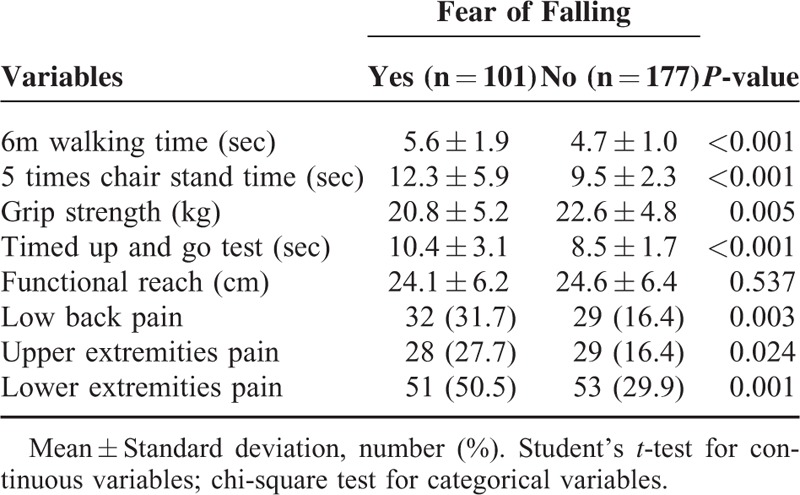
Comparison of Physical Performance or Pain Between Subjects with Fear of Falling and Those Without

Multivariate logistic regression was performed to assess factors affecting fear of falling in the elderly women (Table 4). The Hosmer–Lemeshow test showed no significant difference between observed and predicted prevalence. After adjustment for age, poor physical performance (longer 6-m walking time, longer 5-time chair stand time, weaker grip strength, and longer TUG) and pain (low back pain, upper extremity pain, and lower extremity pain) were significantly associated with fear of falling. No association was found between fear of falling and functional reach. Additional adjustment for BMI, comorbidities, falls in previous year, and cataracts did not alter these associations.

**TABLE 4:**
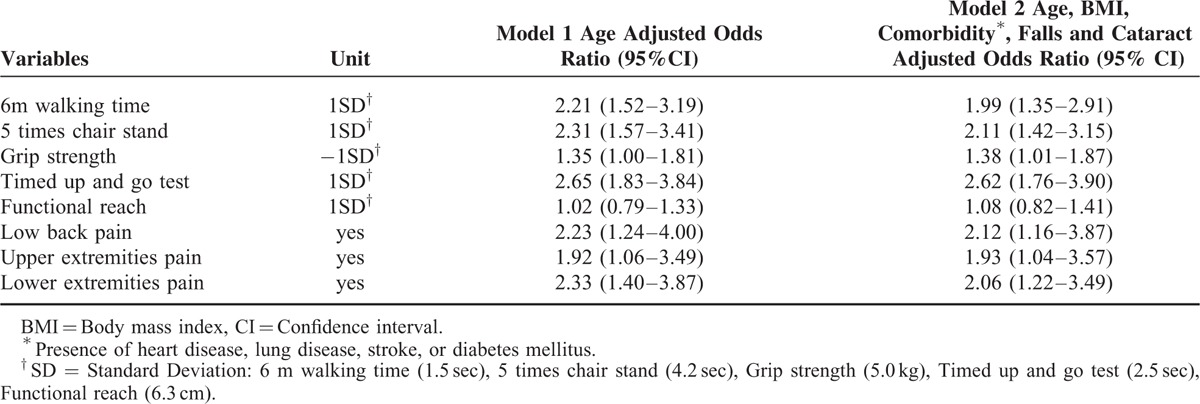
Independent Associations with Fear of Falling (n = 278)

## DISCUSSION

We found that 36% of the elderly women in the present study had fear of falling. This prevalence increased with age, which was similar to previous reports.^21–23^ The prevalence of fear of falling among community-dwelling elderly women differs between studies with reports of 35% in the United States,^5^ 66% in the Netherlands,^23^ and 84% in Korea.^24^ Variations in the prevalence of fear of falling may be due to differences in population characteristics, such as age distribution, fall history, frailty, or culture.

We found that fear of falling was associated with poor physical performance in walking time, chair stand time, grip strength, and TUG. Park et al^15^ reported that elderly with fear of falling had a lower Short Physical Performance Battery score, longer TUG, and weaker grip strength. Rochat et al^13^ reported that fear of falling was associated with reduced gait performance. A decline in physical performance leads to deterioration in the ability to cope with physical challenges on a daily basis and may increase one's fear of falling regardless of previous experience with falls.^15^

Previous studies have shown a relationship between fear of falling and decreased muscular strength (decreased grip strength and increased time to perform 5 chair stands).^12,13^ Brouwer et al^25^ reported that elderly with fear of falling had curtailed their daily activities, which could lead to diminished muscle function. Elderly with muscle weakness may avoid performing activities of daily living, which may further encourage muscle weakness. Muscle weakness may result in perceived vulnerability or loss of confidence when performing everyday activities.^25^ Although the relative timing of events (declines in strength, activity reduction, and fear of falling) is not known because of the cross-sectional design of the present study, the relationship between these events could be reciprocal rather than unidirectional.

Several studies reported the relationship between fear of falling and pain.^26,27^ We found that upper extremity, lower extremity, and low back pain were significantly associated with fear of falling. Gillespie et al^17^ reported that fearful subjects were more likely to report low back pain and lower extremity arthritis. Pain may increase older adult's risk of developing fear of falling.^18^ Clinicians working with older adults with pain should consider assessing fear of falling and, if necessary, intervene if they identify an individual at risk.^18^

Several studies reported that impaired balance is associated with fear of falling.^28,29^ Although Kressig et al^16^ reported the association between fear of falling and functional reach,^30^ we found no association between them. Compared with our subjects (mean functional reach were 24.1 cm in women with fear of falling and 24.6 cm in women without fear of falling, respectively), subjects in the study of Kressig et al may be more frail; mean functional reach were 10.34 cm in the fearful group and 11.85 cm in the not fearful group, respectively. The association between fear of falling and functional reach may be found among older adults transitioning to frailty.

Comorbidity, visual impairment, and experiencing a fall were reported to be associated with fear of falling.^7,10,11,21^ Thus, we conducted logistic regression analysis, adjusting for these variables. Because we used cataracts as a surrogate for visual impairment, all visual impairments may not have been identified. Our results might underestimate the associations with fear of falling.

There are limitations to this study. First, in this cross-sectional analysis, a causal relationship was not necessarily shown by our results. Longitudinal studies are required to establish causal relationships between fear of falling and physical performance or pain. Second, the subjects in this study were recruited from community-dwelling residents who voluntarily attended a health examination. Women with poor health were not examined, which might have affected the results. Third, data on depression or cognitive function was not available in our study. These limitations may contribute to the underestimate of the associations. Fourth, the present results were obtained from only Japanese women; therefore, it is not possible to extrapolate the results to men or to other ethnicities.

We showed that poor physical performance and pain were associated with fear of falling in community-dwelling Japanese elderly women. Maintenance of physical functioning and pain management may be important for elderly women with fear of falling.
